# Genomic Approaches to Uncovering the Coevolutionary History of Parasitic Lice

**DOI:** 10.3390/life12091442

**Published:** 2022-09-16

**Authors:** Kevin P. Johnson

**Affiliations:** Illinois Natural History Survey, Prairie Research Institute, University of Illinois, 1816 South Oak Street, Champaign, IL 61820, USA; kpjohnso@illinois.edu

**Keywords:** phylogenomics, population genomics, Phthiraptera, endosymbionts, mitochondrial genomes, cryptic species

## Abstract

**Simple Summary:**

New sequencing technologies have now made it possible to sequence entire genomes for a diversity of life on earth. Parasites comprise nearly half of all species. Lice are one important group of parasites of birds and mammals, including humans. Genome sequencing approaches have been applied to this group of parasites to uncover patterns of diversification. These patterns can be compared to the patterns of diversification in their hosts. Key findings from these studies have revealed that parasitic lice likely originated on birds and then switched to mammals multiple times. Within groups of birds and mammals, the evolutionary trees of lice match those for mammal hosts more than those for birds. Genomic approaches have also revealed that individual birds and mammals harbor distinct populations of lice. Thus, these new techniques allow for the study of patterns of diversification at a wide variety of scales.

**Abstract:**

Next-generation sequencing technologies are revolutionizing the fields of genomics, phylogenetics, and population genetics. These new genomic approaches have been extensively applied to a major group of parasites, the lice (Insecta: Phthiraptera) of birds and mammals. Two louse genomes have been assembled and annotated to date, and these have opened up new resources for the study of louse biology. Whole genome sequencing has been used to assemble large phylogenomic datasets for lice, incorporating sequences of thousands of genes. These datasets have provided highly supported trees at all taxonomic levels, ranging from relationships among the major groups of lice to those among closely related species. Such approaches have also been applied at the population scale in lice, revealing patterns of population subdivision and inbreeding. Finally, whole genome sequence datasets can also be used for additional study beyond that of the louse nuclear genome, such as in the study of mitochondrial genome fragmentation or endosymbiont function.

## 1. Introduction

High-throughput sequencing technologies now make it possible to explore the coevolutionary history of hosts and parasites at the genomic level. In addition to revealing the mysteries of parasite genomes, genomic approaches can be used to study parasite evolutionary history at a variety of scales over space and time. Shotgun sequencing approaches that generate reads of a whole organism (and associated organisms) can also be leveraged for additional study beyond a parasite itself. These approaches are changing rapidly, so summaries of recent progress in this field can provide a helpful orientation regarding how these techniques can be applied to various topics. In light of the recent advances in high-throughput sequencing technologies, here I review the application of genome sequencing approaches to study the coevolutionary history of a major group of parasitic insects.

**Figure 1:**
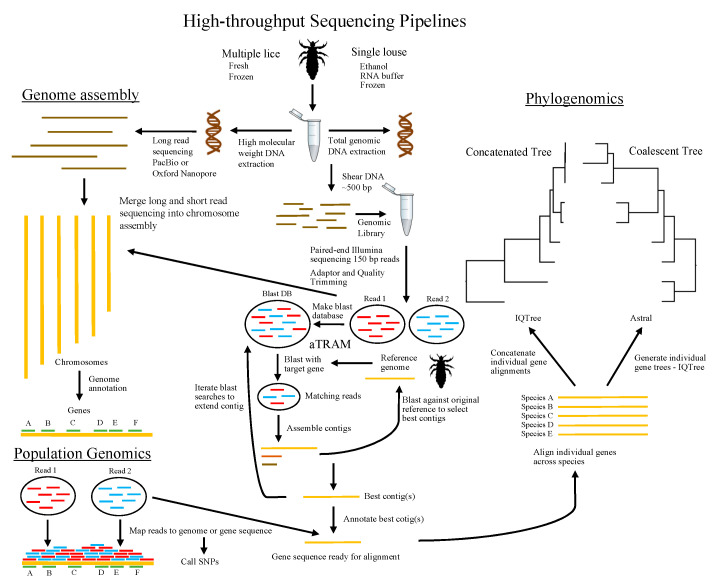
Overview of high-throughput sequencing pipelines as they have been applied to parasitic lice in fields of genome assembly, phylogenomics, and population genomics. Louse image credit Kosta Mumcuoglu (vectorized by T. Michael Keesey) Creative Commons Attribution-ShareAlike 3.0 Unported license.

Parasitic lice (Insecta: Phthiraptera) are wingless ectoparasites of birds and mammals, including humans [1,2]. Sucking lice (Anoplura), parasitic to mammals, have piercing-sucking mouthparts and exclusively feed on blood. Some lice of birds with chewing mouthparts (Ischnocera) feed almost exclusively on feathers. Other groups of chewing lice (Amblycera and Trichodectera) have more variable diets. These parasites spend their entire lifecycle on the body of the host, and they glue their eggs to the hairs or feathers of their hosts. Lice have been a model system for studies of host defenses [3], adaptations to host defense [4], and cophylogenetic studies comparing host and parasite phylogenies [5]. Such studies are now being integrated with information available in the genomic era, opened up by high-throughput sequencing. The term “genome sequencing” is often applied to also include whole genome assembly and annotation. However, genome (or high-throughput) sequencing approaches also have many additional applications that do not include the steps of whole genome assembly and annotation [6]. Many of these varied approaches and applications have recently been applied to parasitic lice (Figure 1).

## 2. Whole Genome Sequencing, Assembly, and Annotation

Some of the early challenges in applying genome sequencing technologies to parasitic lice were the quantity and quality of DNA needed for library preparation and sequencing [6]. Lice are among the smallest insects, and extraction from an individual louse typically yields only 10–100 ng of DNA. Extracting the high-molecular-weight DNA needed for long-read sequencing from such small insects was also an early challenge. Not surprisingly, the first major genome sequencing studies were of species that could be readily kept in culture in high numbers.

The first such louse to have its genome fully assembled and annotated was the human body (head) louse *Pediculus humanus* [7]. The first sequencing was conducted from a culture of human body lice that could be reared on rabbits. However, the head and body ecotypes of *Pediculus humanus* are now believed to be a single species that exhibits phenotypic plasticity [8,9] and differences in microbiome composition [10] depending on conditions. The body louse form lays its eggs in clothing and feeds only one to five times per day [8]. This body louse ecotype also transmits epidemic typhus, trench fever, and louse-borne relapsing fever, so it is of serious medical concern [8]. The head louse ecotype glues its eggs (nits) to hair and feeds much more frequently, from four to ten times a day. This ecotype is not known to readily transmit diseases, which may be related to bacterial suppression [10]. The head louse ecotype has evolved resistance to many common insecticides, and it has become more widespread in recent years, generating economic costs in control [8].

Given the medical and economic importance of this parasite species to humans, it was only the second hemimetabolous insect to have a full genome sequence at the time of publication [7]. In addition, the genome size of the human body louse is among the smallest for any insect at only 110 Mbp, making complete genome sequencing relatively cost-effective. At the time, next-generation sequencing technologies were only just beginning to be developed and the sequencing of this genome was conducted with Sanger technology. Although this genome contains many of the genes that are widespread among insects, the number of genes involved in environmental sensing and detoxification are substantially reduced.

The only other published louse genome sequenced, assembled, and annotated to date is that of the pigeon wing louse (*Columbicola columbae*) [11]. This feather-feeding louse of pigeons (*Columba livia*) has been a model for ecological studies of the interactions between birds and feather lice and is also available in culture. Unlike the sequencing for the human body louse, sequencing for the pigeon wing louse employs high-throughput sequencing technologies, including a combination of Oxford Nanopore, Illumina, and Hi-C technologies. At 208 Mbp total, this genome was found to be almost twice the size of the human body louse genome, albeit still relatively small compared to that of most insects. The assembly of the pigeon wing louse was also greatly improved over the human body louse, with nearly fully assembled end-to-end scaffolds of the 12 chromosomes. Like the human body louse, the genome of the pigeon wing louse has a reduced number of protein-coding genes compared with other insects, including reductions in the number of opsin genes, odorant receptors, and detoxification pathways. The assembly of the pigeon wing louse also showed no evidence of centromeres, in contrast to that for the human body louse.

Although sequencing for both the human body louse and pigeon wing louse have relied on having a large number of individuals available for DNA extraction, new extraction and library preparation techniques may now make it possible to sequence and assemble an entire genome from a single louse [6]. Since many lice are difficult to obtain and generally occur at a low abundance on an individual host (typically < 10 individuals), the ability to sequence the genome from single individual lice will be key to representing the full diversity of these parasites with genome-scale data.

## 3. High-Throughput Shotgun Sequencing

While it may now be possible to sequence and assemble an entire genome from a single individual, much of the historically preserved material available in genetic resource collections has been preserved in ethanol, which fragments DNA. In addition, the costs associated with PacBio or other long-read sequencing platforms using these approaches may be prohibitive for a large number of species. One alternative is to leverage Illumina technology to cost-effectively sequence short (~150 bp) paired-end reads at a coverage of about 30–50X across the louse genome. Given the size of louse genomes, this approach is currently around an order of magnitude less costly than long-read sequencing approaches, such as PacBio, from a single individual. Illumina sequencing libraries can also be prepared from single individuals, even those with fragmented DNA, since Illumina paired-end libraries typically use only around 400–500 bp fragment insert sizes. This Illumina shotgun high-throughput sequencing approach has now been extensively applied to a wide diversity of parasitic lice across varied taxonomic scales. The magnitude and scope of the genomic data now available has led to marked advances in knowledge about louse evolutionary history and population genomics.

Several approaches can be applied to shotgun short-read genome sequences to develop a phylogenomic dataset, typically focused on mining sequences of a predetermined set of single-copy nuclear ortholog genes. The first approach is to produce a draft genome assembly from these short-read data. While suboptimal for total de novo genome assembly, these short reads can still be assembled into a draft assembly. While not expected to be as highly contiguous as assemblies that incorporate long-read data (see above), assembly techniques that can increase the contiguity of assemblies, even from short-read data only, are continuing to be developed [12]. The contigs and scaffolds in this draft assembly can then be annotated for genes of interest for phylogenomics. One such test of this approach in lice annotated the Basic Universal Single-Copy Ortholog (BUSCO) gene set [13] using the BUSCO pipeline, and it was generally successful at recovering from 1168 to 1623 of the 1658 BUSCO gene sequences [12]. One advantage of this approach is that it is not necessary to obtain a reference gene set from within the taxon of interest, since BUSCO uses a diverse set of already existing reference genomes for the annotation. While this approach has not been widely applied in louse phylogenomics or insect systematics more broadly, it is relatively straightforward and can be relatively computationally feasible. Another approach (ALiBaSeq) uses the alignment of bait sequences to a draft assembly to identify and stitch together genes of interest [14], although this approach has not yet been applied to lice.

When a set of reference gene sequences is available, a second approach that could be used in this case is read mapping assembly. Read mapping is a commonly used approach in genomics, and it can be applied to develop a phylogenomic dataset if sequences from a set of phylogenetically useful genes, such as single-copy orthologs, are known for a species closely related to those under study. Since read mapping approaches rely on sequence similarity at the DNA level, one drawback of this approach is that the reference taxon must be very closely related to the other taxa included in the study. Species that are highly divergent from the reference will not generally produce high-quality read mapping results [6]. Another drawback is that genomic regions with insertions and deletions (indels) are difficult to resolve by read mapping, although specialized tools for indel calling now exist [15]. Read mapping is relatively computationally efficient, because all genes can be simultaneously mapped from a genomic sequencing library.

Finally, a third approach that alleviates the need for both a de novo whole genome assembly and highly similar reference sequences is the use of a target restricted assembly method [16,17,18]. This approach relies on blast searches (typically tblastn for highly divergent taxa) of individual protein-coding orthologs against a short-read library to identify reads that may belong to a particular gene. These reads are then locally assembled from only the matching reads using de novo techniques. The resulting contigs are then compared to the original target reference to identify those belonging to the gene of interest. Direct annotation techniques, such as Exonerate [19], can then be used to annotate the start and stop of genes, as well as intron-exon boundaries. Software for this approach, automated Target Restricted Assembly Method (aTRAM), has been developed [17,18] and widely employed in louse phylogenomics [20], generating datasets of hundreds to thousands of nuclear protein-coding genes. Because this approach can use tblastn searches (i.e., amino acid reference), even reference sequences that are highly divergent from the species of interest can be used for the blast and local assemblies [17]. Reference sequences of *Pediculus humanus* (either 1107 or 2395 gene target sets) have been successfully used with the aTRAM approach across all lice [21,22] and even as references for the more highly diverged free living bark lice [23]. One drawback of this approach is that as the number of targeted genes increases, the computation time increases because each gene involves a separate blast search and assembly. However, this approach is extremely flexible across a wide variety of genomic markers from single-copy protein-coding orthologs [17] to ultra-conserved elements (UCEs) [18] and mitochondrial genomics [24]. Many studies of louse phylogenetics have successfully employed the aTRAM approach across a wide variety of taxonomic scales (Table 1).

### 3.1. Origins of Parasitism

One of the main questions concerning the evolutionary history of parasitic lice is whether they have a single common ancestor (i.e., are a monophyletic group). Early morphological and molecular studies generally provided convincing evidence that parasitic lice (historically classified as the Order Phthiraptera) are phylogenetically derived from within free-living bark lice, rendering the traditional Order for free-living bark lice (Psocoptera) paraphyletic [51,52]. Most evidence pointed to a single family of bark lice (Liposcelididae) as being the sister taxon of parasitic lice. Some bark lice are associated with mammal and bird nests [53], feeding on fungi and other organic debris. There are also many records of bark lice in the pelage and plumage of mammals and birds [53], suggesting a transition from a commensal association to a parasitic one by lice [51].

Several morphological characteristics unite parasitic lice into a single group, but many of these are losses or reductions in characteristics, such as loss of wings, loss of ocelli, and reduction in mouthparts, which could be related to their parasitic habit and possibly convergent [51]. Thus, the monophyly of parasitic lice had been difficult to fully demonstrate from morphological characteristics alone [54]. The authors of one study employing sequences of the nuclear ribosome 18S gene concluded that parasitic lice were actually not monophyletic, with the suborder Amblycera being more closely related to the bark louse family Liposcelididae than to other parasitic louse groups [55]. This result implies that parasitism evolved twice. More recent studies have investigated the origins of parasitic lice by using 2395 ortholog gene sequences from transcriptomes across hemipteroid insects [25] (including bark lice and parasitic lice) or by combining ortholog genes from transcriptomes and the same 2395 genes by the aTRAM assembly of Illumina genomic reads [23] across a broader diversity of bark lice and parasitic lice. These studies [23,25] have now provided strong evidence that parasitic lice are derived from a single common ancestor (i.e., are monophyletic), refuting the evidence from 18S alone, and are deeply embedded within free-living bark lice, being the sister taxon of Liposcelididae. Based on this combination of prior morphological and phylogenomic evidence, current classification recognizes bark lice and parasitic lice together in a single insect order (Psocodea), and parasitic lice retain the name Phthiraptera, though now at the rank of Infraorder within Psocodea [23].

Given the convincing evidence that parasitism evolved only once in this group, a question arises: when did this occur? Modern parasitic lice only occur on birds and mammals. However, many non-avian dinosaurs are now known to have feathers or feather-like structures. Is it possible that the origins of parasitism are old enough, predating the Cretaceous–Paleogene (K–Pg) mass extinction event, that non-avian dinosaurs may have had lice? The only convincing fossil louse (*Megamenopon rasnitsyni*) is an excellent compression fossil from 44 million years ago that has characteristics of being a bird louse from the louse family Menoponidae, appearing to have fossilized feather barbs in its crop [56]. This fossil, together with the timing of highly supported codivergence events of lice with primates, has been used to provide a time scale to phylogenies based on molecular data, ranging from Sanger-based [57] to phylogenomic studies [21,25]. All these studies agree that the origins of parasitism by lice predates the K–Pg mass extinction event of the non-avian dinosaurs at 66 mya, suggesting the possibility that these dinosaurs could have hosted lice given that they were also endothermic like birds and mammals. However, there is a broad range of estimates for the specific timing of this origin (92–171 mya) and some conflict as to whether most of the radiation of modern lice occurred before or after the K–Pg boundary, 66 mya. Studies using genomic data with high taxon sampling within parasitic lice [21] have generally converged on the result that most of this radiation was after the K–Pg boundary, coincident with the diversification of major bird and mammal lineages after the extinction of the non-avian dinosaurs. One assumption of these studies is the incorporation of a best estimate of the maximum root age, which in most cases must be inferred from imperfect fossil evidence or prior studies. Variation in root age assumptions can have a substantial impact on the estimates of the timing of subsequent diversification [22]. Hopefully further discoveries of louse fossils, combined with further evidence on the timing of louse and host codivergence events, will enable more accuracy and precision regarding the timescale over which the diversification of parasitic lice occurred.

### 3.2. Major Host Transitions

Recent phylogenomic studies on the higher level relationships of lice and their origins have also clarified the major groups within lice. Historically, parasitic lice were classified [51] as sucking lice (Anoplura) and three groups of chewing lice (Amblycera, Ischnocera, and Rhynchophthirina). Sucking lice (Anoplura) only occur on mammals. Members of Amblycera and Ischnocera widely occur on both birds and mammals, with some families within these major groups restricted to one host group or another. Only three species of lice are in the group Rhynchophthirina [1], and these have chewing mandibles at the end of a long rostrum that they use to pierce the thick hides of their mammalian hosts (elephants, wart hogs, and bush pigs). Given that the major groups of lice have a mix of bird and mammal hosts, three major questions arise: what was the ancestral host of lice, how many transitions between bird and mammal hosts have occurred, and in what direction have these transitions occurred?

Two recent phylogenomic studies of lice have addressed this question. The authors of the first [21] used aTRAM assembled sequences of 1107 nuclear ortholog genes and taxon sampling across all the major groups of lice. This study revealed that the family of mammal-infesting lice, Trichodectidae, traditionally placed within Ischnocera, was actually more closely related to the two other groups of lice exclusive to mammals (Anoplura and Rhynchophthirina), rendering Ischnocera paraphyletic. Based on this and further work [23], the Trichodectidae have been removed from Ischnocera and placed in their own Parvorder, Trichodectera, at the same rank as Anoplura, Rhychophthirina, Amblycera, and remaining Ischnocera. These results shed new light on the number of predicted host-switching events between birds and mammals, suggesting the occurrence of four of these events [21]. However, based on this tree, the direction of these events could not be reconstructed with confidence.

A follow-up study using 2395 genes and expanding taxon sampling within the larger mammal louse clade (particularly Anoplura and Trichodectera) and within Amblycera provided much stronger evidence, with a 100% relative maximum likelihood that the ancestral host of all parasitic lice was a bird [22]. Subsequently, there were four major host-switching events from birds to mammals. Two of these occurred within Amblycera (to Australian marsupials and to South American marsupials and rodents), and one occurred within Ischnocera (to Madagascan lemurs). The final switch was to the ancestor of the major mammal louse clade (Trichodectera, Rhynchophthirina, and Anoplura). Interestingly, all of the earliest diverging genera in this mammal louse clade are hosted by members of the mammalian group Afrotheria (e.g., elephants, hyraxes, and elephant shrews), suggesting that the common ancestor of Afrotheria was the original host for this group of mammalian lice. Other groups of placental mammals then acquired their lice through host-switching out of Afrotheria [22].

In addition to major transitions between birds and mammals, phylogenomic datasets of parasitic lice can also be used to study patterns of host association within these major groups of lice. For example, within avian feather lice, a major clade (Heptapsogasteridae) on tinamous, a group of Neotropical partridge-like birds related to ratites (ostriches, rheas, kiwis, etc.), has long been recognized based on unique morphological features [58]. This clade appears to be an extensive radiation of lice on tinamous, with some tinamou species hosting up to 10 genera from this clade [1].

Based on the unique morphology of these lice and the ancient position of tinamous among birds, Heptapsogasteridae was originally considered to be a “basal” lineage of feather lice [59]. This group consists of members with the “body” ecomorph form, living in the feathers of the body and escaping from preening by burrowing in the down of the body feathers. However, tinamous also host single genera representing the other three ecomorphs: head (*Pseudophilopterus*), wing (*Pseudolipeurus*), and generalist (*Tinamatoecola*) [60]. These genera have previously not been placed with Heptapsogasteridae body lice based on morphology, and they also differ in the way in which they escape host preening defenses. Generally, avian head lice have a rounded body and triangular head, with strong mandibular muscles used for gripping onto feather barbs of the head feathers to avoid being removed by scratching (birds cannot preen their head with the bill). Avian wing lice have a long and slender body and escape host preening by inserting between the feather barbs of the wing [4]. Generalists are of intermediate form and move around the body to escape preening.

Recent phylogenomic studies of tinamou lice using 1107 ortholog genes assembled from genome sequencing reads have revealed that members of Heptapsogasteridae are in a highly derived position within avian feather lice [21,29]. Furthermore, a study based on the same 1107 ortholog gene set that also sampled a broad representation of tinamou louse genera, including members of all four ecomorphs, revealed that not only did members of Heptasogasteridae form a monophyletic group but also that the head, wing, and generalist lice formed a clade that was among the sister taxon to Heptapsogasteridae [30]. This study revealed an extensive in situ radiation of the feather lice of tinamous into divergent ecomorphs and also body louse genera. Among the closest relatives of tinamou lice were other lice from South American endemic families, the hoatzin (Opisthocomidae) and trumpeters (Psophiidae). These relationships suggest that tinamous may have been the source for the host-switching of their feather lice to other South American birds, leading to additional parasite diversification [30].

### 3.3. Cophylogenetics

In addition to resolving higher level phylogenies of lice with more confidence than ever before, phylogenomic datasets using genome sequence data are providing resolution at a variety of taxonomic scales across the tree of lice. These trees can then be compared to those for their bird and mammal hosts using cophylogenetic approaches. Fundamental questions are whether some groups of lice codiverge with their hosts more than others and whether there are underlying ecological and evolutionary mechanisms that explain these differences.

Comparisons of the deep time phylogenomic trees of avian and mammalian louse genera with the phylogenies for birds and mammals appear to point to differences in the pattern of co-diversification in the two groups. Although molecular dating evidence points to the diversification of feather lice occurring after the K–Pg boundary, when most of the major bird lineages also diversified, there is little evidence that they extensively co-diversified with birds at deeper timescales [29]. Out of 36 nodes in a higher level phylogeny of avian hosts, only six (17%) showed evidence of possible codivergence with their feather lice, with much of parasite diversification occurring by host-switching. In contrast, a similar comparison of a tree of mammal lice to their hosts found that 17 out of 30 (57%) nodes in a higher level tree of mammals had an associated codivergence event in their lice [22].

Birds, mammals, and their lice diversified over similar timescales, with major lineages diversifying around the K–Pg boundary [21], so it seems that there may be fundamental biological or ecological differences between the lice hosted by birds and mammals that could explain these differences. In general, birds have higher dispersal capabilities than mammals, and this dispersal may provide more opportunities for host-switching [33]. The sedentary and asocial nature of pocket gophers, for example, has been used to explain the high level of host-specificity and cospeciation in their lice [5]. Another major difference is that avian feather lice have little interaction with the host immune system because they mainly consume feathers, which are inert, while mammalian lice consume blood or sebum and directly interact with the host immune system [61,62]. This interaction in mammal lice may lead to an evolutionary arms race and more coadaptation between mammals and their lice, making host-switching more difficult.

One question is whether these differences between avian and mammalian lice in the role that cospeciation plays in parasite divergence are also apparent at finer taxonomic scales, such as the species level. A substantial number of cophylogenetic studies of the co-diversification patterns of bird and mammal lice have also been conducted at the species level [5,63,64,65,66,67,68,69,70,71,72]; however, in many cases, the phylogenetic trees generated from these Sanger sequencing datasets of a small number of genes are weakly supported. More recent studies that leveraged genome-scale data have now been able to provide high confidence in species-level trees, which has also increased confidence in the resulting cophylogenetic reconstructions (which generally assume that the trees are known).

For example, the authors of a study of the avian feather louse genus *Penenirmus* assembled sequences from 2395 target ortholog genes from genomic reads across 41 species-level samples within the genus [31]. This genus has an interesting pattern of host association that includes two orders of birds: Passeriformes (songbirds) and Piciformes (woodpeckers, barbets, and honeyguides). The phylogenomic trees resulting from analyses of this dataset were highly resolved and supported, as well as generally consistent between concatenated and coalescent phylogenetic approaches. These trees were compared with those for their avian hosts. This comparison revealed that although major groups within *Penenirmus* were associated with host Order or Family, extensive host-switching was also a prominent feature of louse diversification. This host-switching occurred within major biogeographic regions, supporting the idea that host sympatry is an important prerequisite for parasites to switch hosts.

The authors of another species-level study leveraging genomic sequencing for a group of birds (pigeons and doves) and their feather lice (*Columbicola*) also explored the role of biogeography in facilitating host-switching. In addition to assembling a set of 1107 nuclear orthologs for lice using aTRAM, the authors of this study used genomic reads combined with read mapping to obtain sequences from 6363 nuclear genes for their avian hosts, with an alignment length of over 11 million base pairs [33]. Comparisons of host and parasite trees in combination with biogeographic reconstruction revealed that host dispersal was, in several cases, followed by host-switching of lice from resident hosts to newly arriving hosts. In other cases, lice switched from newly arrived hosts to hosts already resident in that biogeographic region. Furthermore, the timing of dove and louse diversification was also estimated using fossil evidence combined with evidence from terminal cospeciation events (i.e., those that occur between pairs of terminal sister taxa). Comparisons of the relative timing of dove and louse diversification in this case revealed that much of dove diversification preceded the radiation of their *Columbicola* feather lice. This resulted in a pattern in which the fraction of parasite speciation events resulting from host-switching declined over time, with cospeciation becoming a more dominant mode of parasite speciation among recently diverged species [33]. The case may be that some dove lineages were free of *Columbicola* wing lice, and these open niches facilitated early host-switching by *Columbicola* when new opportunities of host sympatry, enabled by host dispersal, emerged.

While these phylogenomic studies of avian feather lice have revealed an important role of host-switching in louse diversification, recent phylogenomic studies of mammal lice at the species level have pointed to a more dominant role of the process of cospeciation. A phylogenomic study of seal sucking lice using the 1107 nuclear ortholog gene set revealed that five out of six (83%) louse divergence events could be attributed to codivergence with their seal hosts [41]. Similarly, a phylogenomic study of two genera of lice (*Hoplopleura* and *Neohaematopinus*) from chipmunks (*Tamias*) using the same loci revealed that major clades within each genus were found on closely related species of chipmunks [45]. However, comparisons of louse phylogeny at a deeper level did not reveal as much congruence with chipmunk phylogenies. While a lack of comparable sampling for both lice and chipmunks did not allow the authors of this study to conduct formal cophylogenetic comparisons between chipmunks and their lice, it does appear that cospeciation is a dominant form of parasite divergence, particularly among recently diverged taxa.

The taxonomy and species limits of chipmunk lice in the genera *Hoplopelura* and *Neohaematopinus* are currently unclear, and as currently described, these represent single species across all western chipmunk species. However, based on genetic divergences detected in this study, there are likely to be many undiagnosed species of lice across the diversity of chipmunks [45]. Some recent phylogenomic studies of lice have explicitly explored questions of cryptic species divergence and population structure using extensive sampling.

### 3.4. Cryptic Species and Population Structure

Both the wing (*Columbicola*) and body (*Physconelloides*) lice of small New World ground doves display evidence of cryptic species, as well as variation in host specificity. Genomic sequencing data have been applied to examine these patterns in both of these louse genera across the same group of host species (doves in the genera *Metriopelia*, *Claravis*, *Uropelia*, and *Columbina*). In these studies [34,35], genomic sequencing reads were mapped against reference sequences for each louse genus from 1107 loci assembled using aTRAM. This read mapping approach has the advantage that variable sites can be resolved as single nucleotide polymorphism (SNP) loci, rather than being resolved as only a single base through consensus assembly techniques. Thus, sites that are heterozygous can be identified for each individual and used in both phylogenomic and population genomic approaches.

For the wing lice (*Columbicola*) of small New World ground doves, the widespread species *Columbicola passerinae* was found to be composed of two cryptic species [34]. These cryptic species were each found on several host species, such that neither of them were host-specific. In contrast, species of *Columbicola* found on Andean ground doves in the genus *Metriopelia* were each specific to a single host species. These two cryptic species within *Columbicola passerinae* evidenced from nuclear loci also corresponded to deep divergences in mitochondrial sequence, with an approximately 12% uncorrected sequence divergence. One of the cryptic species showed population structure according to both host and biogeographic regions, while the other did not.

For the body lice (*Physconelloides*) of small New World ground doves, the widespread species *Physconelloides eurysema* was found to be composed of five cryptic species [35]. Some of these appeared to be host-specific, while others were not. These cryptic species were detectable using either nuclear or mitochondrial gene sequences. Similar to the case of *Columbicola*, the lice found on Andean ground doves in the genus *Metriopelia* were host-specific. One cryptic species of *Physconelloides eurysema* (#3) was geographically widespread across six different host species. Within this species, genetic variation was significantly associated with biogeography more so than host species.

In general, the wing lice of doves are more capable of dispersal than body lice because wing lice make use of hitch-hiking (phoresis) on hippobosid flies and body lice do not [73]. Comparing patterns between the wing and body lice of small New World ground doves across the same host species revealed that body lice generally showed more codivergence with their hosts and more evidence of population structure than wing lice [35]. This might be expected given their dispersal differences. In addition, body lice were found to have a significantly lower heterozygosity than wing lice [35], thus suggesting more inbreeding that may have resulted from less dispersal among host individuals. Furthermore, inferred patterns of introgression in this system [36] revealed that wing lice (*Columbicola*) had more evidence of introgression between species than body lice (*Physconelloides*), again suggesting a role for dispersal in creating opportunities for hybridization. Together, these studies show the power of applying genomic sequencing techniques to study the divergence and population structure of parasitic lice.

### 3.5. Population Genomics

Genomic approaches can also be applied to study population structure and patterns of genetic variation within a single species of louse. One intriguing study used genomic resequencing to investigate an unusual case of secondary contact in the blood-feeding louse *Polyplax serrata* (Anoplura) on the mouse *Apodemus flavicollis* in Europe [48]. The mouse harbors two divergent mitochondrial lineages that are currently broadly mixed across Europe, presumably as a result of dispersal from separated glacial refugia after glacial retreat. Their lice, *Polyplax serrata*, also have two deeply divergent mitochondrial lineages, but these are geographically segregated, coming together in a narrow hybrid zone. The examination of nuclear SNPs revealed a similar pattern of strong differentiation across a hybrid zone, though with some admixture between nuclear and mitochondrial genetic variation at the narrow hybrid zone. The most likely explanation for this pattern was that during glaciation, the lice became different species with post-zygotic isolation while their hosts did not. Thus, after range expansion, the mouse hosts freely interbred across a contact zone while their lice did not, creating a zone of parasite turnover.

Another avenue for examining the population genomic structure of parasitic lice is to compare the geographic structures of louse populations to those of their hosts. For permanent parasites, host dispersal is expected to lead to parasite dispersal because hosts carry their parasites with them. In addition, lice have a high degree of vertical transmission between parents and offspring, so patterns of louse dispersal between host individuals are expected to mirror the breeding pedigrees of their hosts in many ways. However, lice may also disperse to unrelated host individuals through other interactions, and this may lead to the mixing of parasite populations in a way that does not directly reflect patterns of host genetic structure.

These patterns have been investigated [39] using genome-scale sequence data for ptarmigans in Alaska and two genera of their parasitic lice (*Goniodes* and *Lagopoecus*). For avian hosts, double-digest restriction-associated DNA sequences (ddRADSeq) were obtained from across the genome of two species of ptarmigans (*Lagopus lagopus* and *L. muta*). For their associated lice, short-read genome wide sequencing was performed, and reads were mapped to 1107 single-copy ortholog reference sequences to identify SNP loci. For the genus *Goniodes*, one louse species *G. lagopi* was found across both host species. Within *Goniodes lagopi*, there was only slight evidence of structure between populations on the two host species. Beyond that, there was little evidence of structure in these louse populations across Alaska. Within *Lagopoecus affinis*, three likely cryptic species were found, each host-specific to a single species of ptarmigan. Within these species, louse population structure was again generally uncorrelated with host population structure.

Because parasitic lice spend their entire lifecycle on the body of the host with limited dispersal opportunities, it might be expected that there is a high level of inbreeding. This inbreeding could lead to highly genetically structured infrapopulations (i.e., the population of parasites on a single host individual). Previous studies using microsatellite data of human lice [74], Galapagos hawk lice [75], and pigeon lice [76] provided evidence for this inbreeding and genetically differentiated infrapopulations. With genomic-scale data, the number of genetic loci that can be employed for such studies can be dramatically increased.

The authors of a study of louse infrapopulations of sucking lice (*Echinophthirius horridus*), sampling two individual lice from 18 individuals of the endangered Lake Saimaa ringed seal, used genomic short-read sequencing combined with read mapping to 1107 nuclear ortholog loci to identify SNPs [42]. Using both phylogenomic and population genomic techniques, the authors of this study found that the two lice from the same seal (i.e., same infrapopulation) tended to cluster together. In addition, even with a sample of just two individuals per infrapopulation, there was significance evidence of inbreeding and population structure for each seal host. The lice also showed strong evidence of geographic structure across the different basins of Lake Saimaa, apparently stronger than the structure in the seals themselves. In addition, estimates of the effective population size for each infrapopulation were correlated between the two louse individuals sampled from each seal, suggesting that each louse harbors genetic variation that is a signature of its own infrapopulation. Seal louse infrapopulations might be expected to show some of the strongest levels of inbreeding among lice because most opportunities for transmission appear to occur over a very short window between mothers and pups when the seals are hauled out on land [77].

## 4. Leveraging Sequence Reads for Additional Study

High-throughput shotgun sequencing libraries contain reads from not only the louse genome themselves but also associated organisms and genomes. These reads could range from the mitochondrial genome to microbial symbionts and even reads from the vertebrate host genome. There is considerable potential to leverage these additional reads for other avenues of study beyond that of the louse nuclear genome itself.

### 4.1. Mitochondrial Genome Organization

The arrangement of mitochondrial genes and the organization of the mitochondrial genome are highly variable among lice and topics of considerable interest. Even in early PCR-based Sanger sequencing studies [78,79,80], lice were known to have highly rearranged mitochondrial genomes compared with other insects. Further studies revealed that the mitochondrial genome of some lice was fragmented into a number of smaller minichromosomes [81,82] instead of all genes being together on a single larger circular chromosome, the typical situation of almost all other animals. For example, in *Pediculus humanus*, the human louse, the mitochondrial genome is arranged across 18 minicircular chromosomes, each harboring one to three mitochondrial genes. These small minicircular chromosomes typically have a non-coding region that is conserved among the minichromosomes [81].

Typical genomic Illumina sequencing read libraries contain a very high coverage of the mitochondrial genome in lice, often comprising over 10 or 100 times more coverage than the nuclear genome. This sequencing has facilitated the more rapid discovery of mitochondrial genome sequences and organization in lice compared with the previous approach of primer-walking with Sanger sequencing. Thus, a number of recent studies have used this high coverage of Illumina reads to assemble the mitochondrial genomes of a variety of lice. The mitochondrial genome appears to be highly fragmented throughout Anoplura, Trichodectera, and Rhynchophthirina [40,47,83], similar to the situation in *Pediculus*, which seems to be a unifying characteristic of these eutherian mammal-infesting groups [26].

Among the Amblycera, many species have a typical single-chromosome mitochondrial genome, although it always appears to be highly rearranged compared with the ancestral insect [26,28,78]. However, some species of Amblycera also have fragmented mitochondrial genomes, with cases of three (*Myrsidea*, *Cummingsia*, and *Laemobothrion*) and seven (*Macrogyropus*) mitochondrial chromosome fragments [27]. In some cases, there is one larger chromosome fragment and two smaller ones (e.g., *Laemobothrion*), while in other cases, the fragments are more similar in size (e.g., *Myrsidea*).

The situation in Ischnocera is even more complex, with some species possessing many minicircular fragments (e.g., *Columbicola* with 17 chromosomes [37]), similar to the situation in *Pediculus*. Other taxa have a single mitochondrial chromosome (e.g., *Campanulotes* [79], *Bothriometopus* [80], and *Falcolipeurus* [32]). Still others have highly variable intermediate levels of fragmentation ranging from 3 to 12 chromosomes [24,82]. Across the phylogenetic tree of Ischnocera, mitochondrial chromosome fragmentation appears to have evolved at least nine times [24]. Fragmentation is also associated with signatures of the relaxed selection on mitochondrial protein-coding genes and a lower AT% bias [24]. Given this high level of variability of mitochondrial genome organization and fragmentation across some groups of lice, there is considerable potential for future studies to uncover specific patterns and processes that might have given rise to the unusual rearranged and fragmented mitochondrial genomes of parasitic lice.

### 4.2. Bacterial Endosymbionts and Microbiomes of Lice

Many lice have a very simplified diet, mainly eating only blood (e.g., Anoplura) or feathers (e.g., most Ischnocera). Like other insects that have a highly specialized diet (e.g., aphids) [84], these lice harbor symbiotic bacteria inside their cells, often in specialized structures called bacteriomes [85]. In insects, such endosymbiotic bacteria are typically maternally transmitted into the eggs. Evidence from the blood-feeding human louse, *Pediculus humanus*, suggests that these endosymbionts provide nutritional supplementation for compounds in short supply in the diet of the louse, such as B vitamins [7].

Shotgun sequences of total DNA from lice often contain sequences of these endosymbiotic bacteria and can be used to assemble the genome of these symbionts. These genome sequences can then be used in phylogenomic studies, as well as in studies of functional genomics, by annotating functional categories of the genes present in the endosymbiont genome. Endosymbiotic bacteria typically have reduced genomes compared with free-living ancestors, losing genes not necessary to the symbiotic relationship. Thus, the function of the endosymbiont can often be deduced by examining gene pathways that are still intact.

The genome of the bacterial endosymbiont, *Candidatus* Riesia pediculicola, of the human louse (*Pediculus humanus*) was assembled at the same time as the genome of the louse itself [7]. This bacterial genome is highly reduced, being only 582 kbp. However, many of the vitamin synthesis pathways (particularly those of B vitamins) are still intact [7], providing corroborating evidence with experiments that demonstrated that B vitamins were crucial to louse development and survival in this species and that this endosymbiont was the source of these B vitamins. Through genomic shotgun sequencing, blood-sucking lice of other primates have also been shown to have bacterial symbionts related to *Riesia*, with similar functional genomic pathways [50]. However, not all species of Anoplura have a *Riesia*-like endosymbiont. The seal louse, *Proechinophthirus fluctus*, harbors a species of *Sodalis* that appears to fill this role [43]. Another anopluran parasitic to rodents, *Polyplax serrata*, harbors a species of *Legionella* [49] (distantly related to known louse endosymbionts) and also has a highly reduced genome of around 539 kbp. Another rodent louse, *Hoplopleura acanthopus*, harbors an endosymbiont with a larger genome (1.6 Mbp) that is within the Neisseriaceae bacterial family [46]. A related bacterium also appears to occur in some samples of *Polyplax serrata*. These cases suggest that endosymbiont replacement may have occurred multiple times during the diversification of Anoplura.

Further evidence of repeated endosymbiont acquisition comes from the bacterial symbionts of feather-feeding lice (Ischnocera). Many species of dove wing lice (*Columbicola*) harbor a *Sodalis* endosymbiont [86]. However, some species harbor different symbiont lineages. The phylogenetic tree of the *Sodalis* endosymbionts of *Columbicola* is star-like [86] and does not reflect the evolutionary history of their louse hosts, as would be expected in a case of long shared co-diversification. Rather, this pattern is expected in a case where a free-living ancestral bacterial lineage repeatedly replaces an existing endosymbiont, resetting the molecular clock, given that endosymbionts tend to evolve much more rapidly than free-living bacteria [86]. The genome of one of these *Columbicola* endosymbionts has been assembled, and it was found to have striking similarities to *Riesia* from *Pediculus humanus*, even though species of *Columbicola* feed on feathers and not blood [38]. It seems likely that feather-feeding lice also rely on their bacterial endosymbionts for vitamin supplementation.

In addition to beneficial endosymbionts, many other bacteria associated with lice can also be discovered through genomic shotgun sequencing. For example, the genome of a species of *Rickettsia* was assembled from the seal louse, *Proechinophthirus fluctus*, in addition to the endosymbiotic *Sodalis* [43]. Based on genome size and content, it did not appear that this *Rickettsia* was endosymbiotic, and it may even have been part of the blood meal of the louse. A study of microbiome variation across infrapopulations of lice on different individual ringed seals revealed significant similarities in the microbiomes of individual lice from the same individual seal [44]. Considerable future potential exists to use shotgun genomic sequences of lice to study both bacterial endosymbionts and microbiomes across the diversity of lice.

## 5. Future Directions

In addition to the topics outlined above, genomic approaches in studies of louse phylogeny and evolution have vast potential to reveal novel patterns and processes. Although phylogenomic studies rely on homologous single-copy orthologs to reconstruct trees, many genes have been duplicated and lost over the course of evolution. Gene and genome assembly and annotation have the capability to identify gene families (i.e., paralogs) and uncover patterns in their evolution. Although much of louse genomic evolution is likely to be a case of loss of genes, one question is whether there are gene families that may have expanded through duplication over the course of louse genomic evolution.

In the field of phylogenomics, there is the potential to incorporate information on allele polymorphism in both the reconstruction of evolutionary trees and the dynamics of effective population size over time. Most phylogenomic studies of diploid organisms ignore heterozygosity and allele polymorphism and make consensus calls at heterozygous sites, reducing the variable position to a single allele. Such sites are also considered a nuisance for genome assembly that should be eliminated if at all possible through the sequencing of inbred lines. However, such polymorphic sites are likely worth considering, particularly in methods that account for incomplete lineage sorting, molecular dating, and estimates of ancestral effective population sizes. Lice appear to have a high density of polymorphic sites across their genome [35,42], with a higher rate of molecular evolution than their hosts [87]. Thus, they could be a test case for methods that incorporate this high level of polymorphism. Assembly methods that account for polymorphism have only recently begun to be developed. In combination with long-read sequencing technologies, these methods have the potential to phase alleles across the entire genome. This will unlock the potential to use the two copies of each chromosome in an individual louse to provide further information on phylogeny, population genomics, and genomic features. Overall, these and many other potential avenues for exploration exist, and they are being unlocked by current and future novel sequencing technologies.

## Figures and Tables

**Table 1:** Overview of high-throughput genome-scale studies of parasitic lice.

Taxon	Topic	References
Phthiraptera	phylogenomics	[21,22,23,25]
	mitogenomics	[26]
Amblycera	mitogenomics	[27,28]
Ischnocera	phylogenomics	[29,30]
	mitogenomics	[24]
*Penenirmus*	phylogenomics	[31]
*Falcolipeurus*	mitogenomics	[32]
*Columbicola*	genome assembly	[11]
	phylogenomics	[33,34]
	population genomics	[35,36]
	mitogenomics	[37]
	microbiomes	[38]
*Physconelloides*	phylogenomics	[35]
	population genomics	[35,36]
*Goniodes*	population genomics	[39]
*Lagopoecus*	population genomics	[39]
Trichodectera	phylogenomics	[22]
*Geomydoecus*	mitogenomics	[40]
Anoplura	phylogenomics	[20,22]
Seal lice	phylogenomics	[41]
	population genomics	[42]
	microbiomes	[43,44]
*Hoplopleura*	phylogenomics	[45]
	microbiomes	[46]
	mitogenomics	[47]
*Neohaematopinus*	phylogenomics	[45]
*Polyplax*	population genomics	[48]
	microbiomes	[49]
*Pediculus*	genome assembly	[7]
	microbiomes	[10,50]

## References

[1] Price R.D., Hellenthal R.A., Palma R.L., Johnson K.P., Clayton D.H. (2003). The Chewing Lice: World Checklist and Biological Overview.

[2] Clayton D.H., Bush S.E., Johnson K.P. (2015). Coevolution of Life on Hosts: Integrating Ecology and History.

[3] Clayton D.H., Lee P.L.M., Tompkins D.M., Brodie E.D. (1999). Reciprocal natural selection on host-parasite phenotypes. Am. Nat..

[4] Clayton D.H., Bush S.E., Goates B.M., Johnson K.P. (2003). Host defense reinforces host–parasite cospeciation. Proc. Natl. Acad. Sci. USA.

[5] Hafner M.S., Sudman P.D., Villablanca F.X., Spradling T.A., Demastes J.W., Nadler S.A. (1994). Disparate Rates of Molecular Evolution in Cospeciating Hosts and Parasites. Science.

[6] Johnson K.P. (2019). Putting the genome in insect phylogenomics. Curr. Opin. Insect Sci..

[7] Kirkness E.F., Haas B.J., Sun W., Braig H.R., Perotti M.A., Clark J.M., Lee S.H., Robertson H.M., Kennedy R.C., Elhaik E. (2010). Genome sequences of the human body louse and its primary endosymbiont provide insights into the permanent parasitic lifestyle. Proc. Natl. Acad. Sci. USA.

[8] Bonilla D.L., Durden L.A., Eremeeva M.E., Dasch G. (2013). The Biology and Taxonomy of Head and Body Lice—Implications for Louse-Borne Disease Prevention. PLoS Pathog..

[9] Amanzougaghene N., Fenollar F., Raoult D., Mediannikov O. (2020). Where are We with Human Lice? A Review of the Current State of Knowledge. Front. Cell. Infect. Microbiol..

[10] Agany D.D.M., Potts R., Hernandez J.L.G., Gnimpieba E.Z., Pietri J.E. (2020). Microbiome Differences between Human Head and Body Lice Ecotypes Revealed by 16S rRNA Gene Amplicon Sequencing. J. Parasitol..

[11] Baldwin-Brown J.G., Villa S., Vickery A., Johnson K.P., Bush S., Clayton D., Shapiro M.D. (2021). The assembled and annotated genome of the pigeon louse *Columbicola columbae*, a model ectoparasite. G3.

[12] Zhang F., Ding Y., Zhu C.-D., Zhou X., Orr M.C., Scheu S., Luan Y.-X. (2019). Phylogenomics from low-coverage whole-genome sequencing. Methods Ecol. Evol..

[13] Waterhouse R.M., Seppey M., Simão F.A., Manni M., Ioannidis P., Klioutchnikov G., Kriventseva E.V., Zdobnov E.M. (2018). BUSCO Applications from Quality Assessments to Gene Prediction and Phylogenomics. Mol. Biol. Evol..

[14] Knyshov A., Gordon E.R.L., Weirauch C. (2021). New alignment-based sequence extraction software (ALiBaSeq) and its utility for deep level phylogenomics. Peer J..

[15] Hasan M.S., Wu X., Zhang L. (2015). Performance evaluation of indel calling tools using real short-read data. Hum. Genom..

[16] Johnson K.P., Walden K.K.O., Robertson H.M. (2013). Next-generation phylogenomics using a Target Restricted Assembly Method. Mol. Phylogenet. Evol..

[17] Allen J.M., Huang D.I., Cronk Q.C., Johnson K.P. (2015). aTRAM—Automated Target Restricted Assembly Method: A fast method for assembling genes from next-generation sequencing data. BMC Bioinform..

[18] Allen J.M., Lafrance R., Folk R.A., Johnson K.P., Guralnick R.P. (2018). aTRAM 2.0: An Improved, Flexible Locus Assembler for NGS Data. Evol. Bioinform..

[19] Slater G.S.C., Birney E. (2005). Automated generation of heuristics for biological sequence comparison. BMC Bioinform..

[20] Allen J.M., Boyd B., Nguyen N.-P., Vachaspati P., Warnow T., Huang D.I., Grady P.G., Bell K.C., Cronk Q.C., Mugisha L. (2017). Phylogenomics from Whole Genome Sequences Using aTRAM. Syst. Biol..

[21] Johnson K.P., Nguyen N.-P., Sweet A., Boyd B.M., Warnow T., Allen J.M. (2018). Simultaneous radiation of bird and mammal lice following the K-Pg boundary. Biol. Lett..

[22] Johnson K.P., Matthee C., Doña J. (2022). Phylogenomics reveals the origin of mammal lice out of Afrotheria. Nat. Ecol. Evol..

[23] de Moya R.S., Yoshizawa K., Walden K.K.O., Sweet A.D., Dietrich C.H., Johnson K.P. (2021). Phylogenomics of parasitic and non-parasitic lice (Insecta: Psocodea): Combining sequence data and exploring compositional bias solutions in Next Generation Datasets. Syst. Biol..

[24] Sweet A.D., Johnson K.P., Cameron S.L. (2022). Independent evolution of highly variable, fragmented mitogenomes of parasitic lice. Commun. Biol..

[25] Johnson K.P., Dietrich C.H., Friedrich F., Beutel R.G., Wipfler B., Peters R.S., Allen J.M., Petersen M., Donath A., Walden K.K.O. (2018). Phylogenomics and the evolution of hemipteroid insects. Proc. Natl. Acad. Sci. USA.

[26] Song F., Li H., Liu G.-H., Wang W., James P., Colwell D.D., Tran A., Gong S., Cai W., Shao R. (2019). Mitochondrial Genome Fragmentation Unites the Parasitic Lice of Eutherian Mammals. Syst. Biol..

[27] Sweet A.D., Johnson K.P., Cao Y., de Moya R.S., Skinner R.K., Tan M., Virrueta Herrera S., Cameron S.L. (2021). Structure, gene order, and nucleotide composition of mitochondrial genomes in parasitic lice from Amblycera (Insecta: *Phthiraptera*). Gene.

[28] Gong S., Xu Y., Xu S., Liang Y., Tian L., Cai W., Li H., Song F. (2022). The Complete Mitochondrial Genome of the Chicken Body Louse, *Menacanthus cornutus*, and Evolutionary Patterns of Extensive Gene Rearrangements in the Mitochondrial Genomes of Amblycera (Psocodea: Phthiraptera). Genes.

[29] de Moya R.S., Allen J.M., Sweet A.D., Walden K.K.O., Palma R.L., Smith V.S., Cameron S.L., Valim M.P., Galloway T.D., Weckstein J.D. (2019). Extensive host-switching of avian feather lice following the Cretaceous-Paleogene mass extinction event. Commun. Biol..

[30] Herrera S.V., Sweet A.D., Allen J.M., Walden K.K.O., Weckstein J.D., Johnson K.P. (2020). Extensive in situ radiation of feather lice on tinamous. Proc. R. Soc. B Boil. Sci..

[31] Johnson K.P., Weckstein J.D., Herrera S.V., Doña J. (2021). The interplay between host biogeography and phylogeny in structuring diversification of the feather louse genus *Penenirmus*. Mol. Phylogenet. Evol..

[32] Nie Y., Fu Y.-T., Zhang Y., Deng Y.-P., Wang W., Tu Y., Liu G.-H. (2021). Highly rearranged mitochondrial genome in *Falcolipeurus lice* (Phthiraptera: Philopteridae) from endangered eagles. Parasites Vectors.

[33] Boyd B.M., Nguyen N.-P., Allen J.M., Waterhouse R.M., Vo K.B., Sweet A.D., Clayton D.H., Bush S.E., Shapiro M.D., Johnson K.P. (2022). Long-distance dispersal of pigeons and doves generated new ecological opportunities for host-switching and adaptive radiation by their parasites. Proc. R. Soc. B Boil. Sci..

[34] Sweet A.D., Boyd B.M., Allen J.M., Villa S.M., Valim M.P., Rivera-Parra J.L., Wilson R.E., Johnson K.P. (2017). Integrating phylogenomic and population genomic patterns in avian lice provides a more complete picture of parasite evolution. Evolution.

[35] Sweet A.D., Johnson K.P. (2018). The role of dispersal in shaping a host–parasite system at multiple evolutionary scales. Mol. Ecol..

[36] Doña J., Sweet A., Johnson K.P. (2020). Comparing rates of introgression in parasitic feather lice with differing dispersal capabilities. Commun. Biol..

[37] Sweet A.D., Johnson K.P., Cameron S.C. (2020). Mitochondrial minicircles in *Columbicola* indicate genome fragmentation occurred repeatedly in parasitic lice. Peer J..

[38] Alickovic L., Johnson K.P., Boyd B.M. (2021). The reduced genome of a heritable symbiont from an ectoparasitic feather feeding louse. BMC Ecol. Evol..

[39] Sweet A.D., Wilson R.E., Sonsthagen S.A., Johnson K.P. (2020). Lousy grouse: Comparing evolutionary patterns in Alaska galliform lice to understand host evolution and host–parasite interactions. Ecol. Evol..

[40] Spradling T.A., Place A.C., Campbell A.L., Demastes J.W. (2021). Mitochondrial genome of *Geomydoecus* aurei, a pocket-gopher louse. PLoS ONE.

[41] Leonardi M.S., Virrueta Herrera S., Sweet A.D., Negrete J., Johnson K.P. (2019). Phylogenomic analysis of seal lice reveals co-divergence with their hosts. Syst. Entomol..

[42] Herrera S.V., Johnson K.P., Sweet A.D., Ylinen E., Kunnasranta M., Nyman T. (2022). High levels of inbreeding with spatial and host-associated structure in lice of an endangered freshwater seal. Mol. Ecol..

[43] Boyd B.M., Allen J.M., Koga R., Fukatsu T., Sweet A.D., Johnson K.P., Reed D.L. (2016). Two bacteria, *Sodalis* and *Rickettsia*, associated with the Seal Louse *Proechinophthirus fluctus* (Phthiraptera: Anoplura). Appl. Environ. Microbiol..

[44] Doña J., Herrera S.V., Nyman T., Kunnasranta M., Johnson K.P. (2021). Patterns of Microbiome Variation Among Infrapopulations of Permanent Bloodsucking Parasites. Front. Microbiol..

[45] Bell K.C., Allen J.M., Johnson K.P., Demboski J.R., Cook J.A. (2021). Disentangling lousy relationships: Comparative phlylogenomics of two sucking louse lineages parasitizing chipmunks. Mol. Phylogenet. Evol..

[46] Říhová J., Batani G., Rodríguez-Ruano S.M., Martinů J., Vácha F., Nováková E., Hypša V. (2021). A new symbiotic lineage related to *Neisseria* and *Snodgrassella* arises from dynamic and diverse microbiomes in sucking lice. Mol. Ecol..

[47] Fu Y.-T., Nie Y., Duan D.-Y., Liu G.-H. (2020). Variation of mitochondrial minichromosome composition in *Hoplopleura* lice (Phthiraptera: *Hoplopleuridae*) from rats. Parasites Vectors.

[48] Martinu J., Stefka J., Poosakkannu A., Hypsa V. (2020). “Parasite turnover zone” at secondary contact: A new pattern in host-parasite population genetics. Mol. Ecol..

[49] Ríhová J., Nováková E., Husník F., Hypsa V. (2017). *Legionella* becoming a mutualist: Adaptive processes shaping the genome of the symbiont in the louse Polyplax serrata. Genome Biol. Evol..

[50] Boyd B.M., Allen J.M., Nguyen N.-P., Vachaspati P., Quicksall Z.S., Warnow T., Mugisha L., Johnson K.P., Reed D.L. (2017). Primates, Lice and Bacteria: Speciation and Genome Evolution in the Symbionts of Hominid Lice. Mol. Biol. Evol..

[51] Lyal C.H. (1985). Phylogeny and classification of the Psocodea, with particular reference to the lice (Psocodea: *Phthiraptera*). Syst. Entomol..

[52] Yoshizawa K., Johnson K.P. (2010). How stable is the “Polyphyly of Lice” hypothesis? A multigene phylogeny of lice and their relatives (Insecta: Psocodea). Mol. Phylogenet. Evol..

[53] Mockford E.L. (1993). North American Psocoptera (Insecta).

[54] Yoshizawa K., Johnson K.P. (2006). Morphology of male genitalia in lice and relatives and phylogenetic implications. Syst. Entomol..

[55] Johnson K.P., Yoshizawa K., Smith V.S. (2004). Multiple origins of parasitism in lice. Proc. R. Soc. Lond. B.

[56] Wappler T., Smith V.S., Dalgleish R.C. (2004). Scratching an ancient itch: An Eocene bird louse fossil. Proc. R. Soc. Lond. B.

[57] Smith V., Ford T., Johnson K.P., Johnson P., Yoshizawa K., Light J.E. (2011). Multiple lineages of lice pass through the K–Pg boundary. Biol. Lett..

[58] Smith V.S. (2001). Avian louse phylogeny (Phthiraptera: Ischnocera): A cladistic study based on morphology. Zool. Soc. Linn. Soc..

[59] Smith V.S. (2000). Basal ischnoceran louse phylogeny (Phthiraptera: Ischnocera: Goniodidae and Heptapsogasteridae). Syst. Entomol..

[60] Johnson K.P., Shreve S.M., Smith V.S. (2012). Repeated adaptive divergence of microhabitat specialization in avian feather lice. BMC Biol..

[61] Ratzlaff R.E., Wikel S.K. (1990). Murine Immune Responses and Immunization against *Polyplax serrata* (Anoplura: Polyplacidae). J. Med Entomol..

[62] James P.J. (1999). Do sheep regulate the size of their Mallophaga louse populations?. Int. J. Parasitol..

[63] Page R., Lee P., Becher S., Griffiths R., Clayton D.H. (1998). A Different Tempo of Mitochondrial DNA Evolution in Birds and Their Parasitic Lice. Mol. Phylogenet. Evol..

[64] Johnson K.P., Adams R.J., Clayton D.H. (2002). The phylogeny of the louse genus *Brueelia* does not reflect host phylogeny. Biol. J. Linn. Soc..

[65] Banks J.C., Palma R.L., Paterson A. (2005). Cophylogenetic relationships between penguins and their chewing lice. J. Evol. Biol..

[66] Johnson K.P., Adams R.J., Page R.D.M., Clayton D.H. (2003). When do parasites fail to speciate in response to host speciation?. Syst. Biol..

[67] Hughes J., Kennedy M., Johnson K.P., Palma R.L., Page R.D.M. (2007). Multiple Cophylogenetic Analyses Reveal Frequent Cospeciation between Pelecaniform Birds and *Pectinopygus* Lice. Syst. Biol..

[68] Light J.E., Hafner M.S. (2008). Codivergence in Heteromyid Rodents (Rodentia: Heteromyidae) and Their Sucking Lice of the Genus *Fahrenholzia* (Phthiraptera: Anoplura). Syst. Biol..

[69] Sweet A.D., Boyd B.M., Johnson K.P. (2016). Cophylogenetic patterns are uncorrelated between two lineages of parasites on the same hosts. Biol. J. Linn. Soc..

[70] Sweet A.D., Bush S.E., Gustafsson D.R., Allen J.M., DiBlasi E., Skeen H.R., Weckstein J.D., Johnson K.P. (2018). Host morphology, parasite morphology, and biogeography influence congruence between host and parasite phylogenies. Int. J. Parasitol..

[71] Catanach T.A., Valim M.P., Weckstein J.D., Johnson K.P. (2018). Cophylogenetic analysis of lice in the *Colpocephalum* complex (Phthiraptera: Amblycera). Zool. Scr..

[72] Catanach T.A., Johnson K.P., Marks B.D., Moyle R.G., Valim M.P., Weckstein J.D. (2019). Two lineages of kingfisher feather lice exhibit differing degrees of cospeciation with their hosts. Parasitology.

[73] Harbison C.W., Clayton D.H. (2011). Community interactions govern host-switching with implications for host–parasite coevolutionary history. Proc. Natl. Acad. Sci. USA.

[74] Leo N.P., Hughes J.M., Yang X., Poudel S.K.S., Brogdon W.G., Barker S.C. (2005). The head and body lice of humans are genetically distinct (Insecta: Phthiraptera, Pediculidae): Evidence from double infestations. Heredity.

[75] Koop J.A.H., DeMatteo K.E., Parker P.G., Whiteman N.K. (2014). Birds are islands for parasites. Biol. Lett..

[76] DiBlasi E., Johnson K.P., Stringham S.A., Hansen A.N., Beach A.B., Clayton D.H., Bush S.E. (2018). Phoretic dispersal influences parasite population genetic structure. Mol. Ecol..

[77] Leonardi M.S., Crespo E.A., Raga J.A., Aznar F.J. (2013). Lousy mums: Patterns of vertical transmission of an amphibious louse. Parasitol. Res..

[78] Shao R., Campbell N.J.H., Barker S.C. (2001). Numerous Gene Rearrangements in the Mitochondrial Genome of the Wallaby Louse, *Heterodoxus macropus* (Phthiraptera). Mol. Biol. Evol..

[79] Covacin C., Shao R., Cameron S., Barker S. (2006). Extraordinary number of gene rearrangements in the mitochondrial genomes of lice (Phthiraptera: Insecta). Insect Mol. Biol..

[80] Cameron S.L., Johnson K.P., Whiting M.F. (2007). The mitochondrial genome of the screamer louse *Bothriometopus* (Phthiraptera: Ischnocera): Effects of extensive gene rearrangements on the evolution of the genome as a whole. J. Mol. Evol..

[81] Shao R., Kirkness E.F., Barker S.C. (2009). The single mitochondrial chromosome typical of animals has evolved into 18 minichromosomes in the human body louse, *Pediculus humanus*. Genome Res..

[82] Cameron S.L., Yoshizawa K., Mizukoshi A., Whiting M.F., Johnson K.P. (2011). Mitochondrial genome deletions and mini-circles are common in lice (Insecta: Phthiraptera). BMC Genom..

[83] Shao R., Barker S., Li H., Song S., Poudel S., Su Y. (2015). Fragmented mitochondrial genomes in two suborders of parasitic lice of eutherian mammals (Anoplura and Rhynchophthirina, Insecta). Sci. Rep..

[84] Dale C., Moran N.A. (2006). Molecular Interactions between Bacterial Symbionts and Their Hosts. Cell.

[85] Sasaki-Fukatsu K., Koga R., Nikoh N., Yoshizawa K., Kasai S., Mihara M., Kobayashi M., Tomita T., Fukatsu T. (2006). Symbiotic Bacteria Associated with Stomach Discs of Human Lice. Appl. Environ. Microbiol..

[86] Smith W.A., Oakeson K.F., Johnson K.P., Reed D.L., Carter T., Smith K.L., Koga R., Fukatsu T., Clayton D.H., Dale C. (2013). Phylogenetic analysis of symbionts in feather-feeding lice of the genus *Columbicola*: Evidence for repeated symbiont replacements. BMC Evol. Biol..

[87] Johnson K.P., Allen J.M., Olds B.P., Mugisha L., Reed D.L., Paige K.N., Pittendrigh B.R. (2014). Rates of genomic divergence in humans, chimpanzees, and their lice. Proc. R. Soc. Lond. B.

